# Individual timing consistency across long‐distance songbird migrations

**DOI:** 10.1002/ece3.11610

**Published:** 2024-09-13

**Authors:** Colin G. Bridges, Saeedeh Bani Assadi, Colin J. Garroway, Kevin C. Fraser

**Affiliations:** ^1^ Department of Biological Sciences University of Manitoba Winnipeg Manitoba Canada

**Keywords:** individual schedules, migration, purple martins, timing consistency

## Abstract

Migration timing in long‐distance migratory birds plays an essential role in individual survival and fitness and is thought to be driven by circannual routines cued by photoperiod with some plasticity to environmental conditions. We examined the individual order of migration timing in purple martins (*Progne subis*), a neotropical migratory songbird that travels between breeding sites throughout eastern North America and nonbreeding sites in Brazil. Migration timing data were collected for 295 different individual purple martins over 9 years using light‐level geolocators deployed at breeding sites across the range. We used linear mixed‐effect models to examine the influence of the rank order of individual departure dates in one season on the rank order of four subsequent migration events while controlling for the effects of breeding latitude, sex, and age. Overall, we found evidence for consistent individual timing that can extend across 8 months and 12,000–24,000 km of migration. Individual rank order of migration timing in purple martins was generally conserved across migrations with consistent timings between fall departure dates from, and spring arrival dates to the breeding site the following year (0.28 ± 0.03, 95% CI 0.22–0.34), as well as at a finer scale across fall migration (0.33 ± 0.05, 95% CI 0.23–0.43), over the stationary nonbreeding period (0.39 ± 0.04, 95% CI 0.31–0.47), and across spring migration (0.03 ± 0.001, 95% CI 0.028–0.032). These results demonstrate that purple martins exhibit consistency in individual migration timing throughout the annual cycle that is likely driven by inherent individual circannual schedules. We additionally found that migration distance played a significant role, as the consistency of individual timing lessened over longer distances. Understanding how individual birds time migrations and if individuals are consistent between events can provide insight into how birds respond to shifts in their environment with climate change.

## Graphical abstract

We used a linear mixed‐effect model to examine the individual rank order of migration of 295 purple martins across their native breeding range. We found evidence for consistent individual timing that can extend across 8 months and 12,000–24,000 km of migration throughout the four yearly migration events with the consistency declining over longer ranges. This demonstrates that individual purple martin migration timing is likely driven by inherent individual circannual schedules.

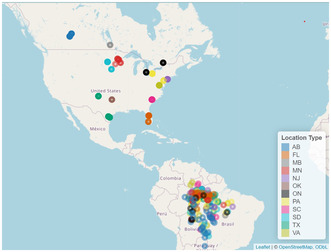

## 1 INTRODUCTION

Animal migration may be driven by a complex interaction between inherent scheduling and external environmental factors, with the yearly migration of birds being one of the most prominent examples (Spiegel et al., 2017). As technology has advanced to enable the detailed tracking of long‐distance migrations (McKinnon & Love, 2018), we can investigate the variation among individuals of a single breeding population experiencing the same external stimuli and if their timing is consistent across migrations (Åkesson & Helm, 2020; Both et al., 2016; Spiegel et al., 2017). Investigating consistent interindividual differences in migration timing variation observed among individuals of the same breeding population (e.g. wherein some individuals may migrate consistently earlier than others) can provide insight into whether these individual schedules are relatively fixed or flexible when faced with environmental variation (Åkesson & Helm, 2020).

In long‐distance migratory songbirds, spring migration timing may be mostly driven by selection on spring departure date for optimal arrival time at breeding sites. Departure timing can be signalled through various environmental cues such as atmospheric pressure, wind speed, rainfall, temperature, or light levels (Cooper et al., 2023; Studds & Marra, 2011), depending on the environment and bird species. Despite flexibility to conditions at departure and en route, timing can be largely repeatable at the individual level suggesting a strong influence of inherent scheduling on overall timing (Both et al., 2016; Fraser et al., 2019; Stanley et al., 2012). Additionally, competition for limiting resources such as nesting space among birds of the same breeding populations may select for earlier migration and consistent yearly timing (Kokko, 1999). Recent genomic analyses suggest that timing phenotypes may be largely prescribed by underlying genomic architecture in key regions (de Greef et al., 2023). However, estimates of the repeatability of individual migration timing do not typically account for across season or year‐to‐year environmental variation that may impact populations more broadly. For example, if migrants experience environmental conditions favouring a later spring departure from the nonbreeding site than the prior year, while they may still be consistent relative to each other, they may all be later than the year before. Therefore, an analysis focussing on individual rank order in migration timing could examine the relative consistency of individuals, when accounting for environmental variation that may influence repeatability (as seen in Fraser et al., 2019).

Purple martins (*Progne subis*) are a Neotropical migratory swallow that breeds in North America and migrates to northern South America over the winter (Brown et al., 2021; Santos et al., 2021). Based upon repeat‐tracking of individual purple martins over 2 years, individuals may vary in their migration timing by up to about 20 days (Fraser et al., 2019), presumably in response to annual environmental variation. However, it is not known if this individual phenotypic plasticity, which allows for a short‐term, rapid adjustment in timing in response to an environmental shift, masks inherent timing upon which selection can act in response to a changing environment (Fox et al., 2019). In pied flycatchers (*Ficedula hypoleuca*), for example, earlier spring migration departure timing was masked by environmental variation birds experienced while en route, such that early departing birds did not arrive earlier at breeding sites (Both, 2010). By looking at the rank order of individuals, we can determine if individual schedules (relative to other individuals) are maintained across migrations, as has been demonstrated in the long‐distance migratory shorebird bar‐tailed godwits (*Limosa lapponica*), which exhibited individual rank order schedules that were maintained across thousands of kilometres of migration (Conklin et al., 2010). Spring departure dates tend to be strongly correlated with spring arrival dates in purple martins (Neufeld et al., 2021), suggesting individual schedules are conserved throughout the migration from the nonbreeding site to the breeding site (Schmaljohann, 2019), but whether there are distinct timing phenotypes within a population that are consistent throughout the annual cycle and across multiple long‐distance migrations has not been explored.

We aimed to examine whether birds exhibit individually consistent chronotypes, which are inherent internal timing processes that vary among individuals, across their spring and fall migrations as compared to other birds in the same population. We infer that individual consistencies across and throughout migrations can be tied to inherent timings but also acknowledge that individual quality may play a role in early or late migration departure. We examined individual timing consistency across four annual timing events: fall departure to fall arrival, fall arrival to spring departure, spring departure to spring arrival, and at the broadest scale, fall departure to spring arrival in the following breeding season. We predicted that if individual inherent schedules are conserved during the course of migration and over periods of breeding and nonbreeding when faced with environmental variation, purple martins would exhibit individual chronotypes that are maintained across their long‐distance migrations (e.g. the earliest birds at one event tend to be the earliest at a later event). However, if conditions during migration disrupt individual schedules, we would expect these chronotypes to diminish as migration distance increases.

Understanding how individual birds time their migration and breeding over multiple years, and if they express consistent individual timing relative to others of the same breeding population, is important for our developing understanding of the ways in which birds may respond or be limited in their timing in response to climate change (Fox et al., 2019; Knudsen et al., 2011), and how this may contribute to long‐term population declines observed in purple martins and other migratory songbirds (Rosenberg et al., 2019).

## 2 METHODS

### 2.1 Data collection

**FIGURE 1:**
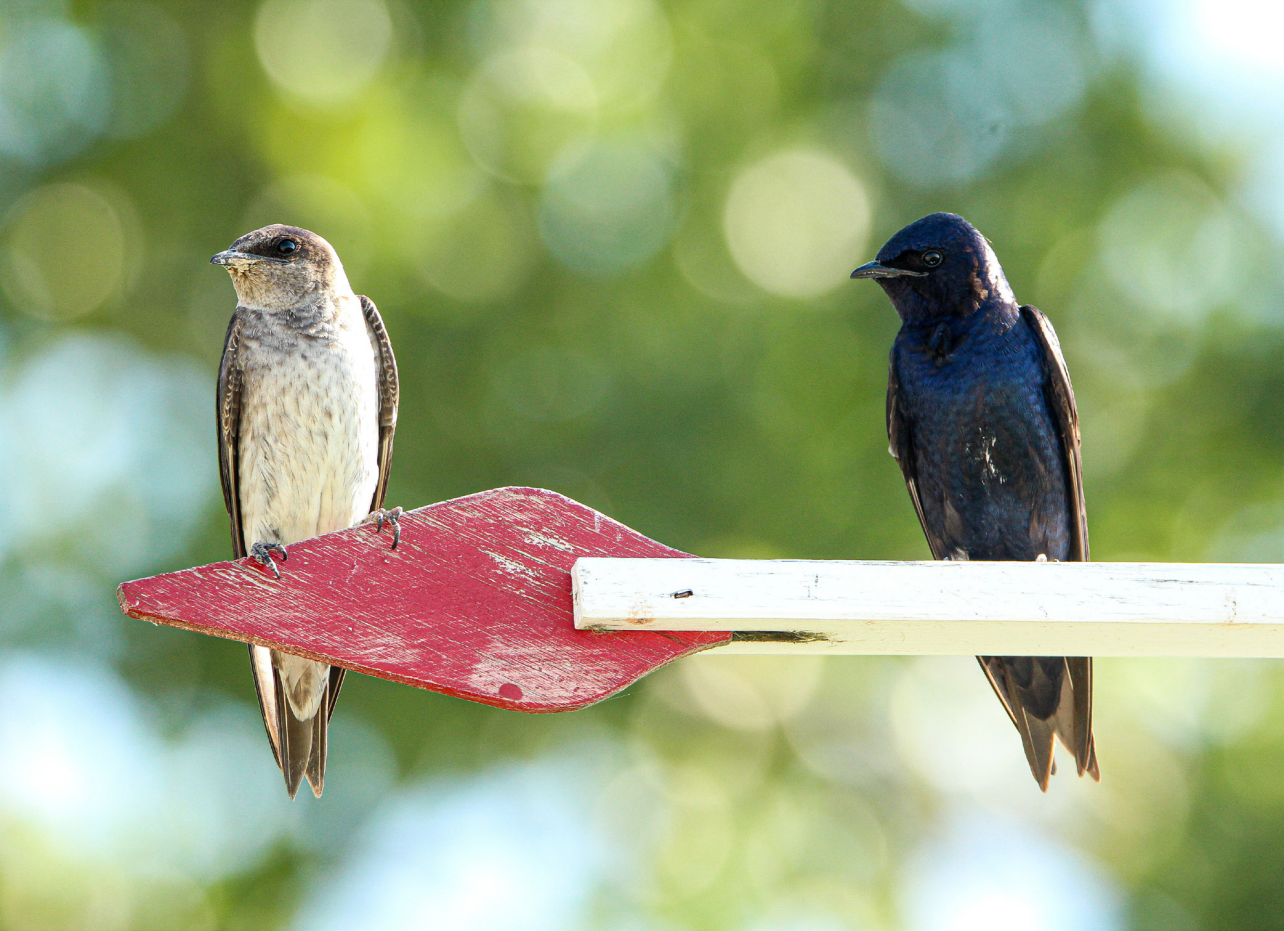
Purple martins perched near a nesting cavity. After second‐year female (left) and after‐second year male (right). Photo: Katie Smith.

**FIGURE 2:**
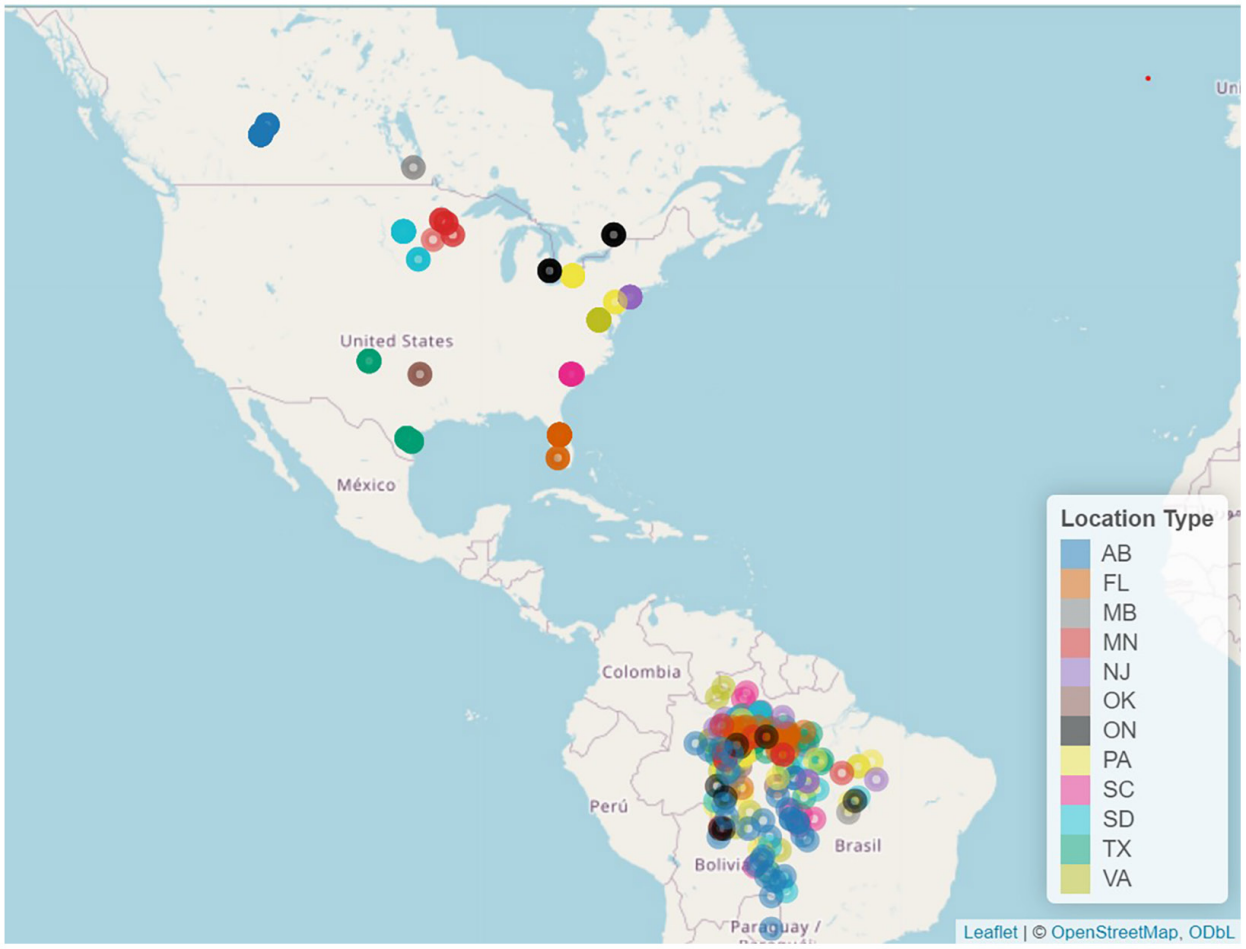
North American breeding sites and communal South American stationary nonbreeding sites. Points are colour coded by breeding site state/province.

Light‐level geolocators (1.6 g; MK10s/12/12 s/14 s/20, British Antarctic Survey) were attached to individual purple martins using a leg‐loop backpack harness (Rappole & Tipton, 1991) when birds were captured at their breeding sites using drop‐door traps. Each bird was aged and sexed based on their plumage (Figure 1; Pyle, 1997). The migration dataset (also used in Neufeld et al., 2021), was created by tagging birds across their eastern North American range and tags were retrieved when birds returned in the following year. This resulted in individual timing data across spring and fall migration and through the breeding and stationary nonbreeding period for 315 individual purple martins originating from 12 breeding sites (Table 1) tracking (Figure 2). We used four key yearly migration dates for each individual: date of fall departure from the breeding site; date of fall arrival at the nonbreeding site in Brazil, date of spring departure from Brazil, and date of spring arrival back to the breeding site the following year.

**TABLE 1:** Latitude and longitude of the 12 breeding sites tracked ranked by closest to furthest distance from the winter site in Brazil.

Breeding sites	Latitude	Longitude
Florida	28.36	−81.59
South Carolina	33.87	−80.18
Oklahoma	33.88	−96.48
Texas	35.04	−101.93
Virginia	38.61	−77.26
New Jersey	40.38	−74.00
Pennsylvania	42.11	−80.14
Ontario	45.35	−75.82
South Dakota	45.59	−98.31
Minnesota	46.14	−93.72
Manitoba	49.73	−97.13
Alberta	52.39	−113.61

Light‐level geolocators use sunrise and sunset to determine the coordinates of a bird at a given time (Conklin et al., 2010). This method may pose issues when tracking bird species who spend prolonged periods in burrows or shade; however, purple martins are aerial foragers, so their behaviour has little impact on light‐data quality (Fraser et al., 2012). Previous estimates of light‐level geolocator error using an earlier subset of these data indicate that estimated positions may differ from known locations by an average of 49–60 km in latitude and 38–48 km in longitude (Fraser et al., 2012). Data were downloaded from retrieved tags using BASTrak software and analysed using the FlightR package in R Studio (R Core Team, 2022, version 4.2.1, Rakhimberdiev et al., 2017). The BAStag package (version 0.1.3, Wotherspoon et al., 2016) was then used to calculate sunrise and sunset times with a threshold set at 32 and the GeoLight package (version 2.0.0, Lisovski & Hahn, 2013) was used to calculate migration timing and daily position during migration and residency periods. Finally, the ggplot2 package (version 3.1.1, Wickham, 2016) was used to analyse and visualise our data (see Neufeld et al., 2021 and Smith & Fraser *in revision* for further details).

### 2.2 Data analysis

To determine whether migration timing was consistent in purple martins, we fit linear mixed models (LMMs) on year‐long migration timing data derived from tracking individuals across all 12 sites from 2007 to 2016 (*n* = 295), pooling all the years to be ranked together. Individual birds with repeat tracking (*n* = 20) over multiple years were removed so only one migration per year, per individual was used in the analysis. We fitted four LMMs, one for each of the four migration timing events around the annual cycle. The first migration event we tested was whether ordering individuals at their fall departure date from the breeding site on spring arrival date was consistent with the order of arrival dates in spring of the following year. Here we examined whether ‘across year rank spring arrival date’ (rank order of individuals) was influenced by the fixed effect of ‘rank fall departure date’ while controlling for the influence of variable climate at different sites and years. A significant effect would suggest individuals have consistent timing schedules that persist over multiple migration events. The other three models targeted migration timing at a finer, within‐season scale. We examined whether the order of birds at their arrival after fall migration (‘rank fall arrival date’) was consistent with the order of their fall departures (‘rank fall departure date’ as a fixed effect). We also examined whether the order of arrival at the beginning of the stationary nonbreeding period (‘rank fall arrival date’) was consistent with the order of departure from the winter site (‘rank spring departure date’) as the fixed effect and whether the rank order of departure from the winter site on spring migration is consistent with the order of arrival at breeding sites (‘rank spring arrival date’).

In all models, we controlled for and investigated the influence of all other factors by assigning three additional fixed effects. The first of these was ‘Latitude’ to test if individuals belonging to lower latitude breeding sites had similar timing consistency when compared to those at higher latitude sites. If latitude significantly affects the timing consistency of individuals, there may be some role for environmental factors altering individual migration schedules as purple martins travel longer distances (i.e. birds breeding at higher latitudes travel the furthest from shared nonbreeding areas). We also included ‘Age’ as a fixed effect, as we may expect younger (second‐year) birds to have generally later timing than older (after second‐year) birds (Morton & Derrickson, 1990). Lastly, we included ‘Sex’ as a fixed effect as we expected different selection pressures on male versus female timing across the annual cycle (Morton & Derrickson, 1990). We also included two random effect variables: ‘Year’ and ‘Site’ to account for variable climate and environmental effects from site latitude and year which could influence migration timing (Fraser et al., 2019). All variables were fitted in LMMs using the lme4 R package (Bates et al., 2015) and a confidence interval of 95% was used for the fixed and response variable. The variance and standard deviation were calculated for the random effects. The models' fit was tested through the MuMIn package (Grabman et al., 2019). *R*‐squared values (marginal *R* ^2^ value was the variance explained by the fixed effects, and conditional *R* ^2^ was the variance explained by the whole model) (Barton, 2019) were used to infer the best model among the selected models. The assumptions of normality and equal variance in our residuals for each model were checked (Zuur et al., 2010).

**FIGURE 3:**
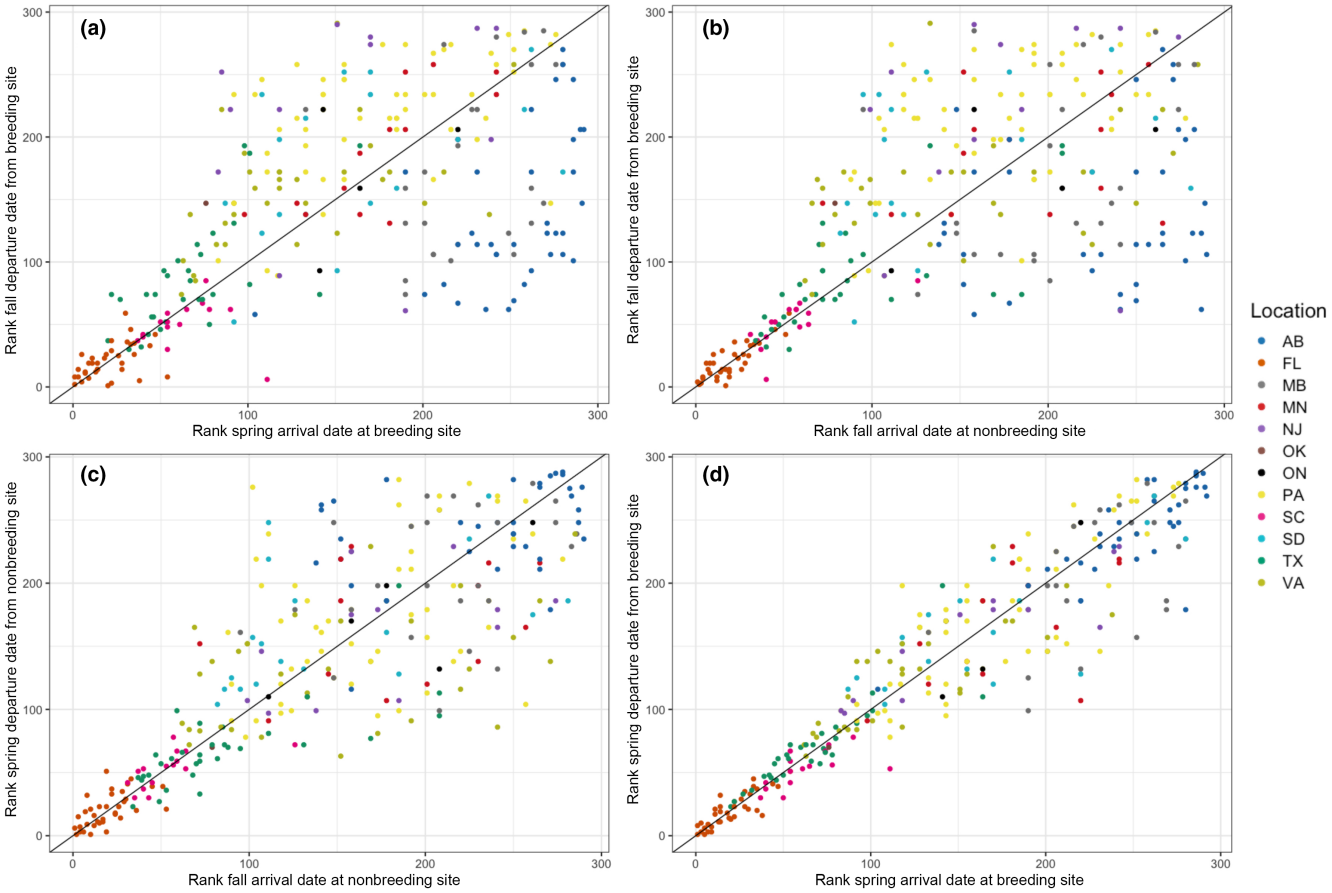
Rank order of individual purple martins compared across seasons of the annual cycle, including (a) rank order of departure from the breeding colony and arrival back to the breeding colony in the following year, (b) rank order of departure from the breeding site and arrival at the nonbreeding range after fall migration, (c) rank order of arrival to the nonbreeding region and departure order in spring, and (d) rank order of spring departure from the nonbreeding area and subsequent arrival order at breeding areas. Lines show 1:1 and individual points are colour‐coded by breeding state/province.

Rank order analyses were done for the four migration events tracking the migration order consistency for all 295 individual birds across the entire North American range. Each individual was scored sequentially in the order of departure or arrival as compared to the entire population and colour coded by site to visualise how migration order may change with increasing breeding site latitude (i.e. migration distance) (Figure 3).

## 3 RESULTS

### 3.1 Across year fall departure and spring arrival dates at the breeding site

The rank order of arrival of individual purple martins at their breeding sites in spring was significantly correlated with the rank order of their fall departure from the breeding site in the previous year. It was also correlated with breeding latitude, and age, but not with sex. The rank order of fall departure from the breeding site had an effect of (estimate ± SE) 0.28 ± 0.03 (95% CI 0.22 to 0.33) (*p* < .001) on the rank order of arrival back at the breeding site the following year. The latitude of the breeding site had an effect of 7.49 ± 0.34 (95% CI 6.82 to 8.16) (*p* < .001) on the rank order of arrival at the breeding site where birds returning to breeding sites at lower latitudes migrated first. The age of the purple martins (ASY) had an effect of −17.28 ± 5.42 (95% CI −27.90 to 6.66) (*p* < .001) on the rank order of spring arrival indicating after‐second year birds migrated earlier than first year birds. The sex (M) of purple martins also had an effect −7.05 ± 4.30 (95% CI −15.48 to 1.38) (*p* = .10) on the spring rank order arrival suggesting that female purple martins tended to migrate earlier than males, but it was not significant.

### 3.2 Fall arrival date at the nonbreeding area

The rank order of fall arrival at the winter site in the Amazon was significantly correlated with the rank order of fall departure from the breeding site and the latitude of the breeding site. The rank order of fall departure from the breeding site had an effect of 0.33 ± 0.05 (95% CI 0.23 to 0.43) (*p* < .001) on the following arrival at the winter site. The effect of the latitude of the breeding site on the fall arrival order 6.00 ± 0.59 (95% CI 4.84 to 7.16) was also significant (*p* < .001). Both age (ASY) −8.47 ± 6.93 (95% CI −22.05 to 5.11) (*p* = .222) and sex (M) −10.13 ± 5.53 (95% CI −20.97 to 0.71) (*p* = .068) had no significant impact on the rank order of arrival at the winter site.

### 3.3 Spring departure date from the nonbreeding area

Rank order of departure from the winter site in the spring was significantly correlated with the rank order of arrival at the winter site in the fall, latitude, and the sex of the individual. Rank fall arrival at the winter site had an effect of 0.39 ± 0.04 (95% CI 0.31–0.47) (*p* < .001) on the rank of departure in the spring. The latitude of the breeding site was 5.54 ± 0.47 (95% CI 4.62–6.46) (*p* < .001) showing a significant impact on the spring departure order. The age of the individuals (ASY) was not significant −8.08 ± 5.94 (95% CI −19.72 to 3.56) (*p* = .175), but sex (M) did have a significant effect on spring departure timing −13.25 ± 4.69 (95% CI −22.44 to 4.06) (*p* = .005).

### 3.4 Spring arrival date at the breeding site

Spring arrival at the breeding site was significantly correlated with all measured fixed effects except sex. The rank order of spring departure from the winter site had a significant effect of 0.03 ± 0.001 (95% CI 0.028–0.032) (*p* < .001) on the rank order of arrival at the breeding sites in the spring. The effect was much smaller than the three other migration events corresponding with a lower variation in migration timing during the spring. The latitude of the breeding site similarly had a significantly positive effect of 0.16 ± 0.02 (95% CI 0.12–0.20) (*p* < .001) on the spring breeding site order of arrival. The age (ASY) of the individual also had a significant effect −0.55 ± 0.17 (95% CI −0.88 to 0.22) (*p* < .001). Sex (M), however, had no significant effect 0.05 ± 0.14 (95% CI −0.22 to 0.32) (*p* = .69) on the rank order of spring arrival at the breeding site.

## 4 DISCUSSION

We show that purple martins had consistent individual arrival and departure schedules in relation to other members of the population (Table 2). These timings persisted throughout the annual cycle, where rank order timing was similar at departure and arrival back to breeding sites dispite an intervening 8 months and 12,000 to 24,000 kilometres of migration. While rank order timing of spring migration was fairly consistent across the entire breeding range, migration order tended to break down for individuals originating from higher latitudes in the other three yearly migration events measured. We therefore infer that longer migration distances may weaken the consistency of individual timing in relation to other birds from the same source population, possibly owing to greater exposure to variable environmental conditions that may disrupt or mask individual timings over longer routes (Both, 2010). Purple martin populations, similar to many other long‐distance migratory bird populations, have plummeted over the past 50 years (Rosenberg et al., 2019). Understanding patterns of timing at broad spatio‐temporal scales can inform our understanding of potential connections between timing and population declines (Both et al., 2006). For example, our findings suggest that individuals with consistantly later chronotypes may be at higher risk when climate change is favouring earlier migration dates.

**TABLE 2:** Summary of linear mixed model (LMM) testing for correlation in rank order of migration timing in purple martins created in *R* using the lme4 package with the marginal *R* ^2^ accounting for the variance of only the fixed effects in the model and the conditional *R* ^2^ accounting for both the fixed and random effects which explain the percent of variation accounted for in this model.

Model		Estimate	SE	95% CI (lower)	95% CI (upper)	m*R* ^2^/c*R* ^2^
Rank order across year spring arrival date	Fixed effects					.79/.83
	Rank order fall departure date	0.28	0.03	0.22	0.34	
	Latitude	7.49	0.34	6.82	8.16	
	Age (ASY)	−17.28	5.42	−27.90	−6.66	
	Sex (M)	−7.05	4.30	−15.48	1.38	
	Random effects	**Variance**		**SD**		
	Year	270.38		16.44		
	Site	70.08		8.37		
Rank order fall arrival date	Fixed effects					.67/.72
	Rank order fall departure date	0.33	0.05	0.23	0.43	
	Latitude	6.00	0.59	4.84	7.16	
	Age (ASY)	−8.47	6.93	−22.05	5.11	
	Sex (M)	−10.13	5.53	−20.97	0.71	
	Random effects	**Variance**		**SD**		
	Year	89.96		9.49		
	Site	278.48		16.69		
Rank order spring departure date	Fixed effects					.79/.83
	Rank order fall arrival date	0.39	0.04	0.31	0.47	
	Latitude	5.54	0.47	4.62	6.46	
	Age (ASY)	−8.08	5.94	−19.72	3.56	
	Sex (M)	−13.25	4.69	−22.44	−4.06	
	Random effects	**Variance**		**SD**		
	Year	15.58		3.95		
	Site	103.82		10.19		
Rank order spring arrival date	Fixed effects					.90/.92
	Rank order spring departure date	0.03	0.001	0.028	0.032	
	Latitude	0.16	0.02	0.12	0.20	
	Age (ASY)	−0.55	0.17	−0.88	−0.22	
	Sex (M)	0.05	0.14	−0.22	0.32	
	Random effects	**Variance**		**SD**		
	Year	0.001		0.04		
	Site	0.35		0.59		

We investigated whether the rank order timing of individuals at a particular migration event was consistent with a future migration event in purple martins and found that individual schedules in purple martins are conserved across the annual cycle. Our results demonstrating consistent individual timing for purple martins, a species of songbird, align with patterns of individual timing previously observed in long‐distance shorebird migration, whereas in the bar‐tailed godwit, individual rank order migration timing was maintained across multiple spring migration events (Conklin et al., 2010). However, in purple martins we found that these individual schedules (relative to others) are also consistent beyond spring migration and extend past long stationary periods of breeding and nonbreeding.

This consistent variation in migration schedules among individuals within the same population may have arisen due to a selection pressure to arrive at their breeding sites during optimal environmental conditions to maximise breeding success and fitness (Kokko, 1999), but the optimal timing of these conditions, such as temperature and food availability, may from vary year to year. Purple martins are one of the earliest spring migrants, possibly owing to strong selection for early arrival when nest cavities are limiting (Neufeld et al., 2021). Year‐to‐year weather at similar calendar dates can be highly variable which may lead to annual variation in optimal arrival timing. In cliff swallows (*Petrochelidon pyrrhonata*), for example, an early cold weather event impacted the survival of early phenotypes, resulting in a shift towards a greater prevalence of later timing phenotypes in the subsequent year (Brown & Brown, 2000). Variable seasonal events such as this may play an important role in differentiating the optimal timing of migration or breeding, resulting in the different individual timings present in purple martin populations. Another driver of variation among the members of a breeding population could be the nesting date as timing set in the nest has been seen to determine future fall departure dates in purple martins (Bani Assadi et al., 2022).

We found that the order of migration timings in individual purple martins relative to others in the same population are also consistent across migrations which could be attributed to three possible explanations. First, direct‐tracking of migratory songbirds has demonstrated high consistency in individual timing in some species (Stanley et al., 2012). Consistency could be favoured if conditions tend to be similar across adjacent years in these short‐lived animals, where what was adaptive in one year may also be successful in a subsequent year. A second nonmutually exclusive explanation may be that timing can be individually consistent owing to the mechanism of timing development in the nest. Laboratory and field studies have shown an ontogenetic effect of daylength during nesting in ‘setting the clock’ for future timing events (Bani Assadi et al., 2022; Bani Assadi & Fraser, 2021; Both, 2010; Coppack et al., 2001; Ouwehand & Both, 2017). In this scenario, early or late nesting results in a corresponding early or late migration timing phenotype. This influence of nest timing on subsequent migration departure may persist across multiple migration events as explained by our rank order results where individual timing relative to other birds of the same population was conserved throughout the annual cycle. However, the inherent individual quality of a bird due to genetic/developmental differences may also independently play a role in determining a bird's migration consistency (Åkesson & Helm, 2020) and warrants further investigation in purple martins. Lastly, carry‐over effects from one season may influence subsequent timing events. For example, the timing of breeding may influence the timing of fall migration, where later nesting results in a later fall departure date (Briedis et al., 2019; Gow et al., 2019). In tree swallows, the effect of nest timing dissolved over the long, winter stationary period, such that there was no effect on spring migration timing and breeding arrival in the subsequent season (Gow et al., 2019). In our study, the rank order of departure after the breeding season and arrival at nonbreeding sites in fall had a higher correlation for birds from lower breeding latitudes and gradually decreased as the migration distance increased. Rather than the influence of carry‐over effects from breeding, this may be better explained by differences in fall stopover duration in martins, where birds from the highest breeding latitudes have longer stopovers (up to several weeks) midway during migration (Loon et al., 2017) which may break down rank order associations evident at other times of year.

It is important to consider how individual timing (relative to others) be maintained across seasons and thousands of kilometres of migration, particularly considering that individual migration timing may vary by up to 20 days in different years (Fraser et al., 2019). If individual migration timing is mostly cued by individual, inherent circannual schedules (de Greef et al., 2023; Gwinner, 1996), then migrating individuals who have started their journey at different dates would be spread out across migratory routes, generally in the order cued by their timing. If conditions across routes were more optimal, such as with earlier spring phenology, the entire cohort may be advanced in their timing in a given year, or delayed if conditions were sub‐optimal. Such a mechanism may explain how individual timing can vary (Fraser et al., 2019), while rank order as observed in our results, remains similar. Further, if environmental conditions slowed leading birds allowing later birds to catch up, we would predict the rank order associations would break down, which may explain why the most northern breeders with the longest migration distances showed weaker rank ordering. While these inferences are highly speculative at this point, they may provide useful framing for further investigation of these themes.

The influence of individual purple martin's age and sex on migration timing varied in effect throughout the year. We found that ASY birds were consistently earlier in migration dates than SY birds throughout the entire year which is consistent with the results of previous studies (Fraser et al., 2019; Morton & Derrickson, 1990). However, the effect of age was only significant from breeding site departure to breeding site arrival in the following year and winter site departure to breeding site arrival. The influence of sex of the purple martins on timing consistency was similarly varied across seasons. Sex had a significant influence on the consistency of timing across the nonbreeding period, being more consistent for females. Our results showed that female purple martins were earlier for all migration events except spring departure from the winter site where males tended to depart first. This result aligns with previous studies of martins that focused on spring migration and found that males tended to initiate migration earlier than females (Morton & Derrickson, 1990; Neufeld et al., 2021), however female timing patterns at spring arrival and at other times of year require further study.

## 5 CONCLUSION

Our results demonstrate that individual migration timing, relative to other individuals of the same breeding colony, was consistent across the annual cycle in purple martins. This pattern carried across a full year and thousands of kilometres of migration, from fall breeding site departure to spring breeding site arrival in the next season. The latitude of the breeding site reduced the timing consistency of purple martins, possibly because individuals heading to breeding sites further north must travel further and thus are exposed to more environmental variability over the course of migration which may disrupt individual timings. Phenotypic plasticity may provide a short‐term flexible response to climate change as individuals can rapidly adjust their migration timing to fit the yearly environmental conditions, but it may also enable individuals with poorly adapted individual migration schedules to persist and reproduce (Fox et al., 2019). Our results demonstrate individual schedules are generally maintained across migration and around the annual cycle with variability likely introduced via plastic responses to environmental conditions encountered en route as migration distance increased (Both, 2010). Since purple martin migration timing phenotypes also correspond to underlying genomic variation (de Greef et al., 2023), timing chronotypes may provide a practical framework for defining conservation units for this declining aerial insectivore in the face of rapid climate change (Miller et al., 2024). Understanding how individual birds time their migrations, and if it is consistent over multiple years, is important to continue to investigate as climate change further alters environmental calendars, which may result in mismatches between bird timings and key resources, contributing to the mass population declines seen among long‐distance migratory birds.

## AUTHOR CONTRIBUTIONS

**Colin G. Bridges:** Conceptualization (supporting); formal analysis (equal); methodology (equal); software (equal); visualization (equal); writing – original draft (lead); writing – review and editing (supporting). **Saeedeh Bani Assadi:** Formal analysis (equal); methodology (equal); software (equal); visualization (equal); writing – review and editing (supporting). **Colin J. Garroway:** Methodology (equal); supervision (supporting); writing – review and editing (supporting). **Kevin C. Fraser:** Conceptualization (lead); data curation (lead); funding acquisition (lead); methodology (equal); project administration (lead); resources (lead); supervision (lead); validation (lead); writing – review and editing (lead).

## FUNDING INFORMATION

The funding provided by the Government of Canada Natural Sciences and Engineering Research Council of Canada NSERC Discovery Program and the University of Manitoba.

## CONFLICT OF INTEREST STATEMENT

There are no competing interests by any of the authors.

## Data Availability

All data used for this paper are available on Movebank.org (Movebank ID 1765768285). https://www.movebank.org/cms/webapp?gwt_fragment=page=studies,path=study1765768285.

## References

[ece311610-bib-0001] Åkesson, S. , & Helm, B. (2020). Endogenous programs and flexibility in bird migration. Frontiers in Ecology and Evolution, 8, 78. 10.3389/fevo.2020.00078

[ece311610-bib-0003] Bani Assadi, S. , & Fraser, K. C. (2021). The influence of different light wavelengths of anthropogenic light at night on nestling development and the timing of post‐fledge movements in a migratory songbird. Frontiers in Ecology and Evolution, 9, 735112. 10.3389/fevo.2021.735112

[ece311610-bib-0004] Bani Assadi, S. , McKinnon, E. A. , Cheskey, E. D. , & Fraser, K. C. (2022). Does hatch date set the clock? Timing of post‐fledging movements for families of a colonially breeding, long‐distance migratory songbird. Journal of Avian Biology, 2022(4), e02766. 10.1111/jav.02766

[ece311610-bib-0005] Barton, K. (2019). MuMIn: Multi‐model inference . R package version 1.42.1.

[ece311610-bib-0006] Bates, D. , Mächler, M. , Bolker, B. , & Walker, S. (2015). Fitting linear mixed‐effects models using lme4. Journal of Statistical Software, 67, 1–48. 10.18637/jss.v067.i01

[ece311610-bib-0007] Both, C. (2010). Flexibility of timing of avian migration to climate change masked by environmental constraints en route. Current Biology, 20(3), 243–248. 10.1016/j.cub.2009.11.074 20116248

[ece311610-bib-0008] Both, C. , Bijlsma, R. G. , & Ouwehand, J. (2016). Repeatability in spring arrival dates in pied flycatchers varies among years and sexes. Ardea, 104(1), 3–21. 10.5253/arde.v104i1.a1

[ece311610-bib-0009] Both, C. , Bouwhuis, S. , Lessells, C. M. , & Visser, M. E. (2006). Climate change and population declines in a long‐distance migratory bird. Nature, 441(7089), 81–83. 10.1038/nature04539 16672969

[ece311610-bib-0010] Briedis, M. , Bauer, S. , Adamík, P. , Alves, J. A. , Costa, J. S. , Emmenegger, T. , Gustafsson, L. , Koleček, J. , Liechti, F. , Meier, C. M. , Procházka, P. , & Hahn, S. (2019). A full annual perspective on sex‐biased migration timing in long‐distance migratory birds. Proceedings of the Royal Society B: Biological Sciences, 286(1897), 20182821. 10.1098/rspb.2018.2821 PMC6408886 30963841

[ece311610-bib-0011] Brown, C. R. , Airola, D. A. , & Tarof, S. (2021). Purple Martin (*Progne subis*), version 2.0. In P. G. Rodewald (Ed.), Birds of the world. Cornell Lab of Ornithology. 10.2173/bow.purmar.02

[ece311610-bib-0012] Brown, C. R. , & Brown, M. B. (2000). Weather‐mediated natural selection on arrival time in cliff swallows (*Petrochelidon pyrrhonota*). Behavioral Ecology and Sociobiology, 47(5), 339–345.

[ece311610-bib-0013] Conklin, J. , Battley, P. , Potter, M. , & Fox, J. (2010). Breeding latitude drives individual schedules in a trans‐hemispheric migrant bird. Nature Communications, 1, 67. 10.1038/ncomms1072 20842198

[ece311610-bib-0014] Cooper, N. W. , Dossman, B. C. , Berrigan, L. E. , Brown, J. M. , Cormier, D. A. , Bégin‐Marchand, C. , Rodewald, A. D. , Taylor, P. D. , Tremblay, J. A. , & Marra, P. P. (2023). Atmospheric pressure predicts probability of departure for migratory songbirds. Movement Ecology, 11, 23. 10.1186/s40462-022-00356-z 37122025 PMC10150475

[ece311610-bib-0015] Coppack, T. , Pulido, F. , & Berthold, P. (2001). Photoperiodic response to early hatching in a migratory bird species. Oecologia, 128(2), 181–186. 10.1007/s004420100652 28547466

[ece311610-bib-0016] de Greef, E. , Suh, A. , Thorstensen, M. J. , Delmore, K. E. , & Fraser, K. C. (2023). Genomic architecture of migration timing in a long‐distance migratory songbird. Scientific Reports, 13(1), 2437. 10.1038/s41598-023-29470-7 36765096 PMC9918537

[ece311610-bib-0017] Fox, R. J. , Donelson, J. M. , Schunter, C. , Ravasi, T. , & Gaitán‐Espitia, J. D. (2019). Beyond buying time: The role of plasticity in phenotypic adaptation to rapid environmental change. Philosophical Transactions of the Royal Society B: Biological Sciences, 374(1768), 20180174. 10.1098/rstb.2018.0174 PMC6365870 30966962

[ece311610-bib-0018] Fraser, K. C. , Shave, A. , de Greef, E. , Siegrist, J. , & Garroway, C. J. (2019). Individual variability in migration timing can explain long‐term, population‐level advances in a songbird. Frontiers in Ecology and Evolution, 7, 324. 10.3389/fevo.2019.00324

[ece311610-bib-0019] Fraser, K. C. , Stutchbury, B. J. M. , Silverio, C. , Kramer, P. M. , Barrow, J. , Newstead, D. , Mickle, N. , Cousens, B. F. , Lee, J. C. , Morrison, D. M. , Shaheen, T. , Mammenga, P. , Applegate, K. , & Tautin, J. (2012). Continent‐wide tracking to determine migratory connectivity and tropical habitat associations of a declining aerial insectivore. Proceedings of the Royal Society B: Biological Sciences, 279(1749), 4901–4906. 10.1098/rspb.2012.2207 PMC3497251 23097508

[ece311610-bib-0020] Gow, E. A. , Burke, L. , Winkler, D. W. , Knight, S. M. , Bradley, D. W. , Clark, R. G. , Bélisle, M. , Berzins, L. L. , Blake, T. , Bridge, E. S. , Dawson, R. D. , Dunn, P. O. , Garant, D. , Holroyd, G. , Horn, A. G. , Hussell, D. J. T. , Lansdorp, O. , Laughlin, A. J. , Leonard, M. L. , … Norris, D. R. (2019). A range‐wide domino effect and resetting of the annual cycle in a migratory songbird. Proceedings of the Royal Society B: Biological Sciences, 286(1894), 20181916. 10.1098/rspb.2018.1916 PMC6367182 30963870

[ece311610-bib-0021] Grabman, J. H. , Dobolyi, D. G. , Berelovich, N. L. , & Dodson, C. S. (2019). Predicting high confidence errors in eyewitness memory: The role of face recognition ability, decision‐time, and justifications. Journal of Applied Research in Memory and Cognition, 8(2), 233–243. 10.1016/j.jarmac.2019.02.002

[ece311610-bib-0022] Gwinner, E. (1996). Circadian and circannual programmes in avian migration. The Journal of Experimental Biology, 199(Pt 1), 39–48. 10.1242/jeb.199.1.39 9317295

[ece311610-bib-0023] Knudsen, E. , Lindén, A. , Both, C. , Jonzén, N. , Pulido, F. , Saino, N. , Sutherland, W. J. , Bach, L. A. , Coppack, T. , Ergon, T. , Gienapp, P. , Gill, J. A. , Gordo, O. , Hedenström, A. , Lehikoinen, E. , Marra, P. P. , Møller, A. P. , Nilsson, A. L. K. , Péron, G. , … Stenseth, N. C. (2011). Challenging claims in the study of migratory birds and climate change. Biological Reviews, 86(4), 928–946. 10.1111/j.1469-185X.2011.00179.x 21489123

[ece311610-bib-0024] Kokko, H. (1999). Competition for early arrival in migratory birds. Journal of Animal Ecology, 68(5), 940–950. 10.1046/j.1365-2656.1999.00343.x

[ece311610-bib-0025] Lisovski, S. , & Hahn, S. (2013). GeoLight – Processing and analysing lightbased geolocator data in R. Methods in Ecology and Evolution, 3, 1055–1059. 10.1111/j.2041-210X.2012.00248.x

[ece311610-bib-0026] Loon, A. V. , Ray, J. D. , Savage, A. , Mejeur, J. , Moscar, L. , Pearson, M. , Pearman, M. , Hvenegaard, G. T. , Mickle, N. , Applegate, K. , & Fraser, K. C. (2017). Migratory stopover timing is predicted by breeding latitude, not habitat quality, in a long‐distance migratory songbird. Journal of Ornithology, 158(3), 745–752. 10.1007/s10336-017-1435-x

[ece311610-bib-0027] McKinnon, E. A. , & Love, O. P. (2018). Ten years tracking the migrations of small landbirds: Lessons learned in the golden age of bio‐logging. The Auk, 135(4), 834–856. 10.1642/AUK-17-202.1

[ece311610-bib-0028] Miller, C. V. , Bossu, C. M. , Saracco, J. F. , Toews, D. P. L. , Rushing, C. S. , Roberto‐Charron, A. , Tremblay, J. A. , Chandler, R. B. , DeSaix, M. G. , Fiss, C. J. , Larkin, J. L. , Haché, S. , Nebel, S. , & Ruegg, K. C. (2024). Genomics‐informed conservation units reveal spatial variation in climate vulnerability in a migratory bird. Molecular Ecology, 33(1), e17199. 10.1111/mec.17199 38018020

[ece311610-bib-0029] Morton, E. S. , & Derrickson, K. C. (1990). The biological significance of age‐specific return schedules in breeding purple martins. The Condor, 92(4), 1040–1050. 10.2307/1368740

[ece311610-bib-0030] Neufeld, L. R. , Muthukumarana, S. , Fischer, J. D. , Ray, J. D. , Siegrist, J. , & Fraser, K. C. (2021). Breeding latitude is associated with the timing of nesting and migration around the annual calendar among purple Martin (*Progne subis*) populations. Journal of Ornithology, 162(4), 1009–1024. 10.1007/s10336-021-01894-w

[ece311610-bib-0032] Ouwehand, J. , & Both, C. (2017). African departure rather than migration speed determines variation in spring arrival in pied flycatchers. Journal of Animal Ecology, 86(1), 88–97. 10.1111/1365-2656.12599 27726147

[ece311610-bib-0033] Pyle, P. (1997). Identification guide to north American birds: Columbidae to Ploceidae. Slate Creek Press. https://works.swarthmore.edu/alum‐books/4499

[ece311610-bib-0034] R Core Team . (2022). R: A language and environment for statistical computing. R Foundation for Statistical Computing. https://www.R‐project.org/

[ece311610-bib-0035] Rakhimberdiev, E. , Saveliev, A. , Piersma, T. , & Karagicheva, J. (2017). FLightR: An R package for reconstructing animal paths from solar geolocation loggers. Methods in Ecology and Evolution, 8(11), 1482–1487. 10.1111/2041-210X.12765

[ece311610-bib-0036] Rappole, J. H. , & Tipton, A. R. (1991). New harness design for attachment of radio transmitters to small passerines (Nuevo Diseño de Arnés para Atar Transmisores a Passeriformes Pequeños). Journal of Field Ornithology, 62(3), 335–337.

[ece311610-bib-0037] Rosenberg, K. V. , Dokter, A. M. , Blancher, P. J. , Sauer, J. R. , Smith, A. C. , Smith, P. A. , Stanton, J. C. , Panjabi, A. , Helft, L. , Parr, M. , & Marra, P. P. (2019). Decline of the North American avifauna. Science, 366(6461), 120–124. 10.1126/science.aaw1313 31604313

[ece311610-bib-0038] Santos, C. O. , Branco, J. M. , Belotti, M. C. T. D. , Abilleira, P. , Siegrist, J. , Fischer, J. , Lima, L. M. , Cohn‐Haft, M. , & Hingst‐Zaher, E. (2021). Distribution and migration phenology of purple martins (*Progne subis*) in Brazil. Ornithology Research, 29(4), 213–222. 10.1007/s43388-021-00071-0

[ece311610-bib-0039] Schmaljohann, H. (2019). The start of migration correlates with arrival timing, and the total speed of migration increases with migration distance in migratory songbirds: A cross‐continental analysis. Movement Ecology, 7, 25. 10.1186/s40462-019-0169-1 31417677 PMC6689889

[ece311610-bib-0040] Spiegel, O. , Leu, S. T. , Bull, C. M. , & Sih, A. (2017). What's your move? Movement as a link between personality and spatial dynamics in animal populations. Ecology Letters, 20(1), 3–18. 10.1111/ele.12708 28000433

[ece311610-bib-0041] Stanley, C. Q. , MacPherson, M. , Fraser, K. C. , McKinnon, E. A. , & Stutchbury, B. J. M. (2012). Repeat tracking of individual songbirds reveals consistent migration timing but flexibility in route. PLoS One, 7(7), e40688. 10.1371/journal.pone.0040688 22848395 PMC3405083

[ece311610-bib-0042] Studds, C. E. , & Marra, P. P. (2011). Rainfall‐induced changes in food availability modify the spring departure programme of a migratory bird. Proceedings of the Royal Society B: Biological Sciences, 278(1723), 3437–3443. 10.1098/rspb.2011.0332 PMC3177634 21450737

[ece311610-bib-0043] Wickham, H. (2016). ggplot2: Elegant graphics for data analysis. Springer‐Verlag.

[ece311610-bib-0044] Wotherspoon, S. , Sumner, M. , & Lisovski, S. (2016). BAStag: Basic data processing for light based geolocation archival tags . R package version 0.1.3.

[ece311610-bib-0045] Zuur, A. F. , Ieno, E. N. , & Elphick, C. S. (2010). A protocol for data exploration to avoid common statistical problems. Methods in Ecology and Evolution, 1(1), 3–14. 10.1111/j.2041-210X.2009.00001.x

